# Engineering Macrophages *via* Nanotechnology and Genetic Manipulation for Cancer Therapy

**DOI:** 10.3389/fonc.2021.786913

**Published:** 2022-01-06

**Authors:** Xiaoling Ding, Xinchen Sun, Huihui Cai, Lei Wu, Ying Liu, Yu Zhao, Dingjingyu Zhou, Guiping Yu, Xiaorong Zhou

**Affiliations:** ^1^ Department of Immunology, Nantong University, School of Medicine, Nantong, China; ^2^ Department of Gastroenterology, The Affiliated Hospital of Nantong University, Nantong, China; ^3^ Department of Clinical Laboratory, Taizhou Peoples’ Hospital, Taizhou, China; ^4^ Department of Clinical Laboratory, The Sixth Nantong People’s Hospital, Nantong, China; ^5^ Department of Immunology, Southeast University, School of Medicine, Nanjing, China; ^6^ Krieger School of Arts & Sciences, Johns Hopkins University, Baltimore, MD, United States; ^7^ Department of Cardiothoracic Surgery, The Affiliated Jiangyin Hospital of Nantong University, Jiangyin, China

**Keywords:** macrophages, bioengineering, nanotechnology, cancer immunotherapy, chimeric antigen receptors

## Abstract

Macrophages play critical roles in tumor progression. In the tumor microenvironment, macrophages display highly diverse phenotypes and may perform antitumorigenic or protumorigenic functions in a context-dependent manner. Recent studies have shown that macrophages can be engineered to transport drug nanoparticles (NPs) to tumor sites in a targeted manner, thereby exerting significant anticancer effects. In addition, macrophages engineered to express chimeric antigen receptors (CARs) were shown to actively migrate to tumor sites and eliminate tumor cells through phagocytosis. Importantly, after reaching tumor sites, these engineered macrophages can significantly change the otherwise immune-suppressive tumor microenvironment and thereby enhance T cell-mediated anticancer immune responses. In this review, we first introduce the multifaceted activities of macrophages and the principles of nanotechnology in cancer therapy and then elaborate on macrophage engineering *via* nanotechnology or genetic approaches and discuss the effects, mechanisms, and limitations of such engineered macrophages, with a focus on using live macrophages as carriers to actively deliver NP drugs to tumor sites. Several new directions in macrophage engineering are reviewed, such as transporting NP drugs through macrophage cell membranes or extracellular vesicles, reprogramming tumor-associated macrophages (TAMs) by nanotechnology, and engineering macrophages with CARs. Finally, we discuss the possibility of combining engineered macrophages and other treatments to improve outcomes in cancer therapy.

## Introduction

Macrophages are a class of immune cells with highly diverse phenotypes and functions. Some macrophages residing in tissues are known as tissue-resident macrophages (TREMs), such as Kupffer cells in the liver and pulmonary macrophages in the lungs. TREMs have a long lifespan, participate in local immune responses, and are essential components to maintain internal homeostasis (1–3). Peripheral monocytes can also be recruited to inflammatory tissues, where they differentiate into macrophages (4). In a typical inflammatory response caused by microorganisms, pathogen-derived molecules known as pathogen-associated molecular patterns (PAMPs), such as lipopolysaccharide (LPS) in bacterial wall, can be detected by macrophages through a group of receptors called pattern recognition receptors (PRRs), which triggers the activation of macrophages (5–7). Activated macrophages can effectively eliminate pathogens by their potent phagocytic activity (5–7). They also recruit immune cells from blood and activate T cell response through antigen processing and presentation, thus playing a key role in both innate and acquired immunity (8–10).

Tumors are often accompanied by a certain degree of inflammatory response (11, 12). Macrophages in tumor tissues are collectively referred to as tumor-associated macrophages (TAMs). Tumor cells frequently overexpress some cytokines, such as macrophage colony-stimulating factor 1 (CSF-1) and monocyte chemoattractant protein-1, (MCP-1), which recruit a large number of macrophages into tumor sites (13). In addition, tumor blood vessels have an irregular structure and abnormal function; they are dilated, leaky, and inefficient at delivering oxygen, which causes hypoxia in tumor tissues (14). Hypoxia in turn induces the expression of vascular endothelial growth factor (VEGF), a key mediator of tumor angiogenesis, but is also a potent macrophage-recruiting cytokine (15). Therefore, macrophages are often the most abundant type of tumor-infiltrating immune cells (16–18). However, the activity of macrophages in tumors is often suppressed; they cannot kill tumor cells efficiently through phagocytosis and overexpress immunosuppressive cytokines, including IL-10 and TGF-β, thereby establishing an unfavorable tumor immune microenvironment (16–18). TAMs also promote tumor cell survival and metastasis and induce drug resistance by secreting growth factors or by direct cell-cell contact with tumor cells (19, 20). Therefore, in many cases, TAMs are protumorigenic, and identifying effective methods to modify TAMs to improve anticancer therapy is of great interest (16–18).

The application of nanotechnology in cancer therapy holds great promise (21, 22). Nanoparticles (NPs) are synthetic structures with a nanoscale dimension and can be generally divided into two categories: organic NPs (i.e., liposomes, polymer micelles) and inorganic NPs (i.e., gold, silver, iron oxide) (23). NPs have been used to deliver a variety of anticancer agents, such as traditional chemotherapeutic drugs (23), targeted drugs (24), and genetic materials [i.e., messenger RNA (25), small interfering RNA (26), and the CRISPR/Cas9 genetic editing system (27)]. Due to their distinctive physicochemical properties, NPs can enhance the delivery of anticancer agents to tumors by both passive and active mechanisms (21, 28). As mentioned above, tumor blood vessels have increased permeability, which allows NPs to pass through the leaky endothelium; meanwhile, due to defective lymphatic drainage, the extraverted NPs can accumulate in the tumor interstitium, leading to an increased local drug concentration, a process known as the enhanced permeability and retention (EPR) (29). However, in many cases, the passive mechanism and EPR are not sufficient (29), and by active targeting strategies, such as ligand-mediated systems (30), stimulus-responsive systems (31), and biological system (32), the efficiency of NP targeted delivery can be improved. For example, most tumors have an increased rate of glycolysis, leading to an acidic environment due to the accumulation of lactic acid. Based on this feature, various pH-responsive systems have been developed (33, 34), which effectively dissociate NPs and decrease their size in low-pH areas (inside the tumors), thereby enhancing their ability to deeply penetrate into tumors (35). Moreover, the NP surface can be modified by ligand molecules that can recognize specific receptors on the tumor cell surface, thus increasing the affinity between tumor cells and NPs, which is critical for effective internalization of NPs by tumor cells (36, 37).

Among various active strategies, biological NP delivery systems are attracting considerable interest (32). NPs can be loaded in cell membranes (CMs), extracellular vesicles (EVs), or even live cells for targeted delivery. Regarding live-cell NP carriers, research mainly focuses on immune cells (38), especially macrophages, as they are superior in their ability to migrate toward tumors. Many studies have demonstrated that NP-loaded macrophages (NPL-Ms) can directionally migrate to tumors and transport the payload to tumor cells, leading to a pronounced antitumor effect (39, 40). Moreover, after reaching tumors, these engineered macrophages can exert additional effects by stimulating anticancer immune responses (24, 41). In this review, we first introduce the origin, differentiation, and function of macrophages as well as the application of nanotechnology in anticancer therapy. Then, we elaborate on the activities, mechanisms, and limitations of the engineered macrophages. Finally, we discuss several new strategies in macrophage engineering and discuss their potential as novel anticancer therapeutics.

## Macrophages Fundamentally Impact the Development of Cancer

**Figure 1:**
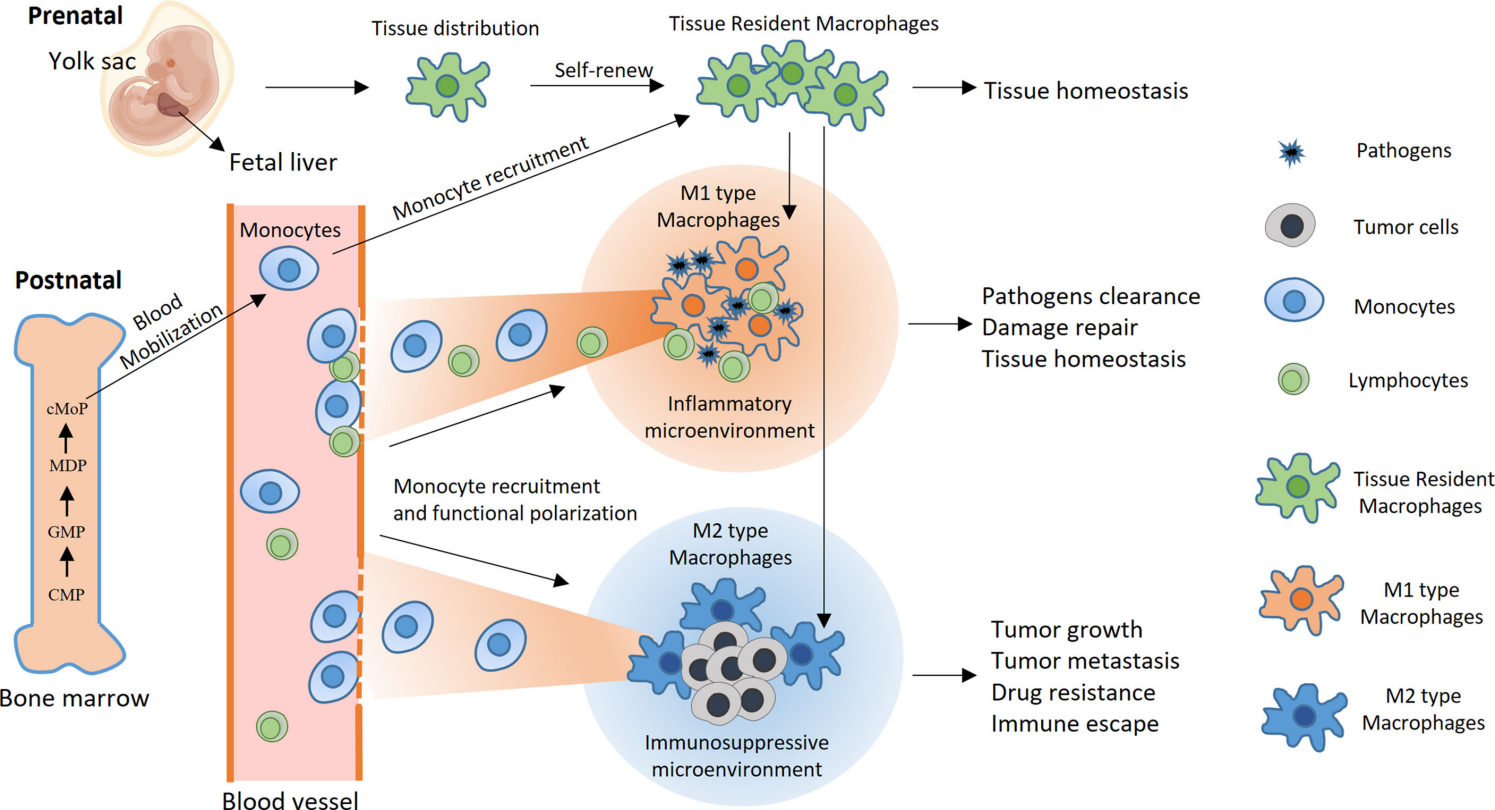
Development, differentiation, and function of macrophages. Under physiological conditions, macrophages are highly versatile and widely present in almost all tissues and organs. Some macrophages that reside in tissues are called TREMs. TREMs originate mainly from yolk sac macrophage progenitors and fetal liver macrophages during embryonic development. After birth, TREMs maintain their number partially through self-renewal and sometimes through the recruitment of monocyte-derived macrophages. Pluripotent hematopoietic stem cells in bone marrow develop into monocytes through multiple stages, including common myeloid progenitors (CMPs), granulocyte-macrophage progenitors (GMPs), macrophage and dendritic cell precursors (MDPs), and common monocyte progenitors (cMoPs). In typical inflammation caused by pathogen infection, monocytes are mobilized from the bone marrow into the blood circulation and subsequently recruited into inflammatory sites, where they differentiate into M1 macrophages and efficiently phagocytose the pathogen. Inflammation also recruits lymphocytes and initiates antigen-specific immune responses with the help of macrophages and dendritic cells, ultimately resulting in pathogen clearance. At the late stage of inflammation, macrophages differentiate toward the M2 type and participate in the tissue repair process, leading to the restoration of internal homeostasis. In contrast, monocytes and TREMs preferentially differentiate toward M2 polarization after they enter the tumor microenvironment, wherein they promote tumor growth and metastasis, mediate resistance to cancer treatments and inhibit antitumor immune responses.

Macrophages are key players in inflammation and participate in the crosstalk between inflammation and cancer development (**Figure 1**). In a typical inflammatory response, macrophages can perform three basic functions: 1) pathogen clearance, i.e., eliminating pathogens through phagocytosis or secreting anti-infective substances (5–7); 2) immune activation, i.e., activating humoral and cellular immune responses by presenting antigens to T cells and modifying the immune microenvironment by releasing a variety of inflammatory factors (8–10); and 3) tissue repair, i.e., releasing factors in the late stage of inflammation that promote angiogenesis, coordinating the functions of a variety of interstitial cells, and mediating the repair of local tissue structure (42, 43). Macrophages can sense environmental stimuli and differentiate into functionally polarized subgroups (44–46), which is usually described as M1 or M2 differentiation, terms that were first used to describe the two functionally opposite statuses of macrophages that are induced *in vitro* (47, 48). Lipopolysaccharide (LPS) and interferon-gamma (IFN-γ) can promote the differentiation of macrophages toward M1 polarization, characterized by high production of nitric oxide (NO), reactive oxygen species (ROS), and a series of proinflammatory cytokines, such as interleukin (IL)-1β and IL-12. M1 macrophages activate T helper type 1 (Th1)-type immune responses and have strong phagocytic and antigen-presenting activities. Hence, they are considered proinflammatory and tumor suppressive (49, 50). In contrast, IL-10, transforming growth factor-beta (TGF-β), and some other immunosuppressive factors, such as IL-4 and IL-13, can induce M2 macrophage differentiation. M2 macrophages participate in the Th2-type immune response, inhibit CD8+ T cell activities, and promote angiogenesis and tissue repair and therefore are believed to be anti-inflammatory and tumor-promoting factors (51, 52). However, recent studies have suggested that the extreme M1/M2 differentiation pattern induced *in vitro* cannot reflect the complex situation *in vivo*. For example, macrophages in the tumor microenvironment often exhibit some characteristics of both M1 and M2 macrophages (44, 53, 54). Although the dichotomy of M1/M2 macrophages is an oversimplification, it is still a meaningful way to describe the functionally poised status of macrophages in certain situations.

There is a close relationship between cancer and inflammation. Tumor growth is often accompanied by a certain degree of inflammation, and the underlying mechanisms are complex (11, 12). For example, chronic viral infection induces constant inflammation and contributes to the development of some types of cancer (55, 56). In addition, tumor blood vessels are often distributed abnormally and have a broken structure, and they cannot meet the oxygen and nutrition requirements of fast-growing tumor cells, resulting in hypoxia and nutrition deficiency within some tumor areas. Consequently, some tumor cells undergo apoptosis or necrosis and release proinflammatory substances, such as adenosine triphosphate (ATP) and high mobility group box 1 (HMGB1), inducing persistent low-grade inflammation and recruiting various immune cells into tumors (57, 58). Macrophages in tumor tissues are collectively referred to as tumor-associated macrophages (TAMs) and are often more numerous than other infiltrated immune cells (16–18). This in itself suggests that macrophages may have a tumor-promoting effect. Numerous studies have demonstrated that tumor cells often express high levels of chemokines, such as GM-CSF, M-CSF, and CXCL12, recruiting many monocytes from the circulation into local tumor sites (15). After entering tumors, monocytes differentiate into mature macrophages, followed by functional polarization toward M2-type TAMs, which is dictated by factors from the immunosuppressive tumor microenvironment. TAMs secrete factors such as CCL22, CXCL1, and PDGF, which bind to corresponding receptors on tumor cells, thereby promoting tumor growth and metastasis, as well as resistance to various cancer treatments (19, 20, 59–61).

In addition, TAMs contribute to the establishment of a deeper immunosuppressive tumor microenvironment by secreting soluble factors and cell-cell contact with other immune cells (54). For example, CCL20 secreted by TAMs recruits regulatory T cells that inhibit the response of effector T cells (62). Moreover, TAMs express low levels of major histocompatibility complex (MHC)-II and costimulatory molecules on the cell surface, which greatly diminishes their ability to stimulate T cells (63). Although TAMs maintain the ability to phagocytose tumor cells to some extent, tumor cells often express high levels of CD47 molecules that bind to signal regulatory protein α (SIRPα) on the surface of TAMs, sending the “don’t eat me” signal and inhibiting the phagocytic activity of TAMs (64). Although many studies have supported the notion that macrophages have tumor-promoting effects, some evidence suggests that macrophages play important antitumorigenic roles in some types of cancers, such as colorectal cancer and early-stage lung cancer (65, 66). More importantly, the functions of macrophages are highly plastic, and their anticancer activities can be reactivated by various means, including macrophage engineering *via* nanotechnology and genetic manipulation, which this review will focus on.

## Nanotechnology in Cancer Therapy

Recently, the application of nanotechnology in cancer therapy has attracted increasing attention (21, 22). NPs travel through the bloodstream to tumor sites, enter the interstitial fluid through the vascular wall *via* passive diffusion, and finally are taken up by tumor cells. However, tumor blood vessels have an abnormal structure, resulting in an uneven distribution of NPs, which often accumulate at the edge of blood vessels, resulting in limited anticancer activity of NPs (29, 67). Active targeting strategies, mainly the use of ligand-mediated systems, stimulus-response systems, and cell-mediated systems, are currently under intensive investigation for their potential to solve the above problem by targeted delivery of NPs to tumor tissues and enhanced tissue distribution and penetration.

The first category of active strategies is the use of ligand-mediated systems. In this strategy, ligands or antibody molecules that recognize biomarkers on tumor cells are present on the shell of NPs, thereby enhancing the interaction between NPs and tumor cells and promoting the transport of NPs to tumor tissues. Targeting biomarkers can be tumor-specific antigens or overexpressed oncoproteins, such as prostate-specific membrane antigen (PSMA) for prostate cancer (68, 69), epidermal growth factor receptor (EGFR) for lung cancer cells (36, 70), and human epidermal growth factor receptor 2 (HER2) for gastric cancer or breast cancer cells (37, 71). However, the outcomes of this strategy to date are often unsatisfactory *in vivo* due to various reasons, such as the high heterogeneity of tumor tissues and the fast clearance of NPs in circulation (72, 73).

The second category is stimulus-response systems. These systems use specific stimulus signals to promote the directional delivery of NPs to tumors and to boost the anticancer activities of NP-carried drugs (31, 74). The signals can be tumor intrinsic, such as an increased glutamine level (75), a decreased pH value (76), and hypoxia (77), or tumor extrinsic, such as a light source (78), a heat source (79), a magnetic field (80), or ultrasound (81). Among them, light-responsive systems may be the most well-studied systems because they can be readily controlled in a spatiotemporal manner, resulting in directional transport, improved tumor penetration and distribution, and controlled release of NP-carried drugs. For more information, please refer to the relevant reviews (82, 83).

The third method involves carrier cells or cell components. As mentioned earlier, the development of many cancers is accompanied by a certain degree of inflammation and immune cell infiltration. Immune cells can sense tumor-derived chemokines and actively move to tumor sites (84, 85). Interestingly, although hypoxia prevents the infiltration of T cells, it stimulates tumor cells to release a large number of macrophage-recruiting factors, such as CCL2, CSF-1, and VEGF, resulting in pronounced enrichment of macrophages in hypoxic tumor regions (15, 86). A series of studies have demonstrated that macrophages can be exploited as cell carriers to actively transport NPs into tumor sites (30, 87), and the following section will introduce the preparation, function, mechanisms, and limitations of NPL-Ms in cancer therapy.

## Engineering Macrophages for NP Delivery in Cancer Therapy

### NP Loading in Macrophages

There are two main sources of macrophages for NP loading. One source is primary macrophages, such as bone marrow-derived macrophages, alveolar macrophages, and peritoneal macrophages. The second source is cell lines, including the mouse macrophage cell lines RAW264.7 and J774A.1 and the human peripheral blood monocyte cell line THP-1 (24, 41, 88–91). NPL-Ms can carry a variety of NPs, including liposomes (92, 93), magnetic NPs (94, 95), polymeric NPs (96, 97), gold (AU) NPs (98–101), and others (102, 103). Because macrophages naturally phagocytose NPs (104, 105), NPL-Ms can be prepared by a simple coincubation method. Li et al. prepared RAW264.7 macrophages loaded with paclitaxel (PTX)-containing NPs. Intravenous injection of NPL-Ms significantly inhibited the growth of a breast cancer model (39). Ibarra et al. prepared mouse bone marrow-derived monocytes and THP-1 cells loaded with polymer NPs, and they showed that NP loading had no significant effect on the viability and function of macrophages, nor did it affect the differentiation of THP-1 cells into macrophages upon stimulation with phorbol 12-myristate 13-acetate (PMA). Moreover, these cells had a stronger NP loading ability after LPS stimulation (96). Electroporation can also be used to prepare NPL-Ms and might be a superior approach for loading easily degradable substances such as nucleic acids or enzyme precursors (106, 107).

NPL-Ms can be exploited for cancer therapy with *in situ* strategies. Because monocytes/macrophages efficiently phagocytose apoptotic bodies, Zheng et al. intravenously injected light-sensitive gold NPs encapsulated by apoptotic bodies, which were quickly engulfed by macrophages, thus generating NPL-Ms *in vivo*. These NPL-Ms effectively migrated to tumor sites and inhibited tumor growth and metastasis in a mouse tumor model (108). Circulating monocytes/macrophages efficiently phagocytose damaged red blood cells (RBCs) *via* the complement-mediated opsonization effect. Based on that, Feng et al. designed a cell relay strategy that allowed monocytes in circulation to preferentially take up NPs. They first prepared NPs coated with artificially damaged RBCs that were used as primary carriers to deliver NPs to macrophages, generating NPL-Ms *in vivo*, which delivered NPs to tumors in a targeted manner, leading ultimately to enhanced anticancer activity in a rat tumor model (109).

In some cases, internalized nanomaterials may negatively affect macrophage function, or the encapsulated drugs in NPs are prematurely dissociated, which may reduce the efficacy of drug delivery or cause systemic toxicity (30). A plausible alternative is the so-called piggybacking method, i.e., binding NPs on cell surface, which has been tested with various cell types, including macrophages (38). Through various techniques that can be largely classified into two categories, noncovalent and covalent, NPs can be attached on cell surfaces without being internalized by the macrophage carrier, and transported to tumor sites (110–116). **Table 1** briefly describes the categories, principles, and mechanisms of major NP delivery methods with live macrophages, and readers are directed to more detailed reviews on this subject (112, 123, 134). **Table 1** also includes the methods of loading NPs in macrophage-derived cell membranes or extracellular vesicles, which will be discussed in the following section.

**Table 1:** NP loading in macrophage-based drug delivery.

Strategies	Categories	Method Descriptions and Mechanisms	REFs
Cell Encapsulation	*In vitro*	Coincubation: cells uptake NPs through phagocytosis or other endocytosis mechanisms. Electroporation: electroporation generates small pores on cell membrane for NPs to entry into cells.	(39, 40, 117–119)
	*In vivo*	Functionalized NPs, NPs tethered on damaged red blood cell (RBC) membranes, or NPs cloaked in apoptotic bodies are engulfed by macrophages to form NP-loaded macrophages *in vivo*.	(108, 109, 120–122)
Surface Binding	Covalent coupling	Modified NPs are coupled to functional groups (i.e., thiol, amine) on cells through various mechanisms, such as maleimide-thiol conjugation and disulfide bond formation. - Complicated procedure, high binding strength, possibly impaired cell integrity	(114, 123, 124)
	Noncovalent binding	Nonspecific adsorption: NPs are attached to outer cell membranes *via* hydrophobic or electrostatic binding. Ligation-mediated binding: NPs modified with ligands or antibodies bind corresponding molecules on the cell surface. - Simple procedure, low binding strength, high cell integrity	(110, 111, 113, 115, 116)
Membrane Coating	–	The procedure may involve the following steps:	(95, 125–128)
		Cell culture: such as tumor cells, RBCs, and immune cells; Isolating the cell membrane by hypotonic treatment; Coating NPs with the cell membrane by various methods, such as coincubation, extrusion, and sonication. - NPs can be camouflaged in homogenous membranes from one cell type or heterogeneous fused membranes from two different cell types.	
EV Loading	–	Extracellular vesicles (EVs) include exosomes and microvesicles derived from various cell types. - The procedure is similar to that of membrane coating but is usually more sophisticated due to the complicated EV isolation procedure. EV-loaded NPs may have an increased ability to pass biological barriers due to their smaller size.	(129–133)

### NPL-M Tumor Site Migration

In a study by Li et al., RAW264.7 macrophages loaded with fluorescent NPs were injected intravenously into normal nude mice, and these NPL-Ms were quickly distributed into the liver and intestine 1-2 h after injection; however, they were almost undetectable after 24 h, indicating fast clearance of the NPL-Ms. In contrast, in nude mice bearing subcutaneous xenograft tumors, the NPL-Ms infiltrated into tumor tissues shortly after injection and resided there for more than 48 h. These findings indicated that NPL-Ms directly migrated toward tumors and had a relatively long half-life in the tumor microenvironment (39). Hypoxia often occurs in tumors and drives the migration of monocytes/macrophages toward tumor sites. This feature renders macrophages a unique type of cell carrier to deliver NPs to hypoxic tumor areas. Choi et al. demonstrated that NPL-Ms carrying gold NPs could migrate toward hypoxic tumor spheres *in vitro* (98). An et al. loaded macrophages with anionic gold nanorods (AuNRs) for hypoxia-triggered photoacoustic (PA) imaging and photothermal therapy (PTT). The results indicated that NPL-Ms directionally migrated to hypoxic tumor sites and provoked significant antitumor effects (135).

Traditional cancer treatments, such as radiotherapy and chemotherapy, also affect the migration of macrophages to tumors. Evans et al. prepared NPL-Ms loaded with hypoxia-activated prodrug NPs and demonstrated that NPL-Ms accumulated in the hypoxic regions of mouse breast tumors. Moreover, the accumulation and anticancer activities of NPL-Ms were more significant when combined with chemotherapy (136). Miller et al. found that radiotherapy increased the intratumoral concentration of NPs in a mouse breast cancer model, which is related to the radiotherapy-induced increase in TAM infiltration. They found that a large number of TAMs accumulated around microvessels after radiotherapy, altered vascular permeability, and elicited dynamic bursts of NP extravasation. Depleting macrophages greatly diminished the effect of radiotherapy on the enrichment of NPs in tumor tissues (122). *In vivo* PET imaging can be performed using macrophages loaded with NPs containing (64)Cu. Based on that, Kim et al. demonstrated that chemotherapy or radiotherapy significantly increased the number of TAMs, thereby increasing the intratumoral NP concentration in mouse tumors (137).

Inducing M1 polarization may enhance the tumor homing activity of macrophages. Peng et al. found that M1 macrophages loaded with DOX-NPs effectively crossed the blood brain barrier (BBB) and exerted a strong inhibitory effect on a mouse glioma model (118). Li et al. prepared macrophages loaded with magnetic NPs. These NPL-Ms exhibited M1 polarization and had significantly enhanced tumor homing and anticancer activities in a mouse breast cancer model. In addition, NPL-Ms improved the tumor immune microenvironment, inhibited local M2 macrophages, and enhanced the antitumor immune response (138).

### NPL-M Drug Release

There are relatively few studies on how NPL-Ms release NPs after reaching tumor tissues. In the piggybacking method (38), membrane-binding NPs are delivered to tumors with the help of macrophages in a targeted manner, and the subsequent release of the drug depends mainly on the design of the NP itself. In terms of NPL-Ms, regardless of whether they are formed *in vitro* or *in situ*, the mechanism of drug release and how the process is controlled remain elusive. Li et al. loaded macrophages with fluorescence-labeled PTX-NPs and then cocultured the macrophages with tumor cells *in vitro*. After 4 h, a fluorescent signal was detected in tumor cells that gradually increased and peaked at 12 h, during which time the signal in macrophages gradually decreased, indicating that the NPs were transferred from macrophages to tumor cells (39). Cells mainly ingest foreign substances through endocytosis, and ultimately, the ingested substances are either degraded or released from cells [please refer to the detailed reviews (139–141)]. Macrophages mainly engulf NPs through phagocytosis and pinocytosis. NPs are not rapidly degraded during intracellular trafficking in macrophages, so the potential adverse effects of the free drug are diminished. In addition, macrophages slowly release ingested NPs, which reduces the consumption of NPs before the macrophages reach tumors. For example, by comparing macrophages loaded with free PTX or PTX-NPs, Li et al. found that 26% of PTX-NPs vs. greater than 50% of free PTX were released before the macrophages reached the tumors (39).

NPL-Ms can transfer NPs or free drugs to tumor cells through other means. For example, tumor cells can interact with and exchange information with other cells through the microtubule network (142, 143). Guo et al. found that M1 macrophages loaded with DOX (DOX-M1) entered mouse tumors and exported DOX to tumor cells through tunneling nanotubes, leading to pronounced tumor cell killing (144). In another study, LPS was anchored to the cell membrane of macrophages loaded with DOX. These macrophages migrated to mouse tumors and rapidly killed tumor cells by transferring DOX to tumor cells through a microtubule network. In addition, cell membrane-anchored LPS induced the differentiation of local TAMs to M1 macrophages and promoted the antitumor immune response (145).

The process of NP release by macrophages is affected by many factors, including the physicochemical properties of NPs, the functional status of macrophages, and the tumor microenvironment. For example, Oh et al. reported that gold NPs with a high-aspect ratio exit macrophages more rapidly but tend to remain in tumor cells longer than those with a low aspect ratio (146). Ikehara et al. found that a mild temperature increase promoted the release of NPs by macrophages (147). In addition, macrophages showed higher drug release efficiency for polymeric or negatively charged copolymer NPs than for liposomal NPs or positively charged copolymer NPs (121, 148, 149). Interestingly, Soma et al. found that IFN-γ stimulation significantly promoted the release of NP-DOX by macrophages (150). During inflammation, activated macrophages release a large amount of cytokines and bioactive substances; therefore, activating macrophages may promote the release of NPs.

### Limitations and Challenges

**Figure 2:**
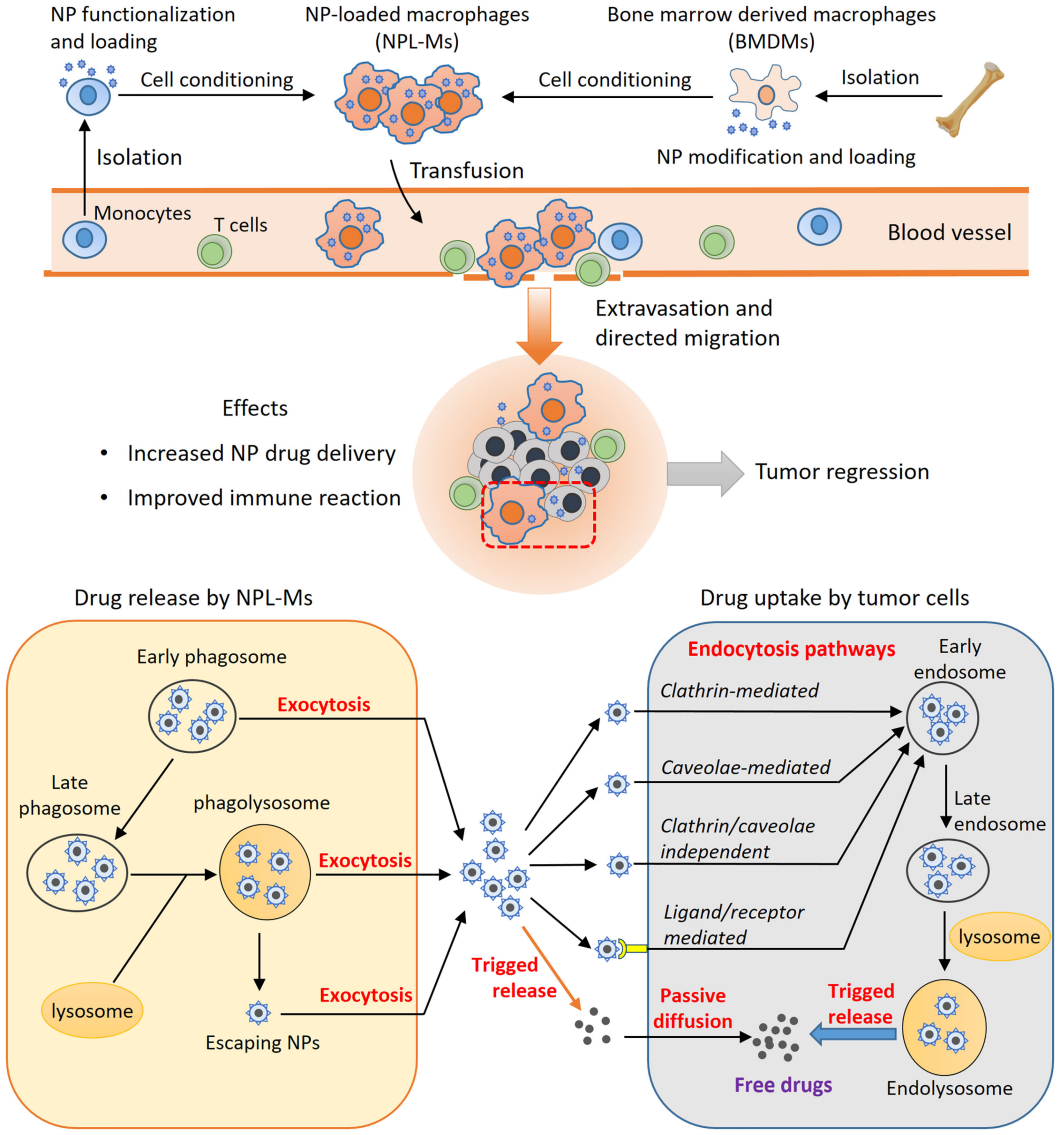
The principles of macrophage-based NP drug delivery. Live macrophage carriers are mainly from peripheral monocytes, bone marrow-derived macrophages, or macrophage cell lines. M1-type macrophage differentiation can be induced, and NPs can be functionalized. After administration, the NPL-Ms migrate to tumors, enhancing drug delivery and anticancer immune responses. The efficiency of this strategy depends on controlled drug release by NPL-Ms and effective drug uptake by neighboring tumor cells. Through exocytosis, NPs recycled from early phagosomes or matured phagolysosomes or NPs that escape from phagosomes can be released through the exocytosis mechanism. Tumor cells uptake NPs through various endocytosis pathways, such as the clathrin-mediated, caveolae-mediated, and clathrin/caveolae-independent pathways. NPs functionalized by surface ligands can be recognized by corresponding receptors on tumor cells and effectively internalized by endocytosis. Consequently, the internalized NPs are sorted into early endosomes, late endosomes, and eventually endolysosomes where NPs can be triggered to release free drugs. Free drugs released from NPs in the intracellular space can enter into tumor cells by passive diffusion.

The concept of using macrophages as drug carriers is not new and has been studied for many years. However, it has not been applied in clinical practice. **Table 2** summarizes some recent preclinical studies using live macrophages for NP drug delivery. In the future, in-depth studies are needed to achieve a better understanding of the complex interaction among NPs, macrophages, and tumor cells. An ideal cell-mediated NP delivery system would have the following five characteristics: 1) an abundant source of cells into which NPs can be loaded efficiently; 2) no significant impairment of cellular function after NP loading; 3) directional migration toward tumors; 4) efficient release of NPs at tumor sites; and 5) effective uptake of the released NPs by tumor cells. Natural evolution has endowed macrophages with powerful phagocytic, migratory and secretory functions. With the advantages provided by nanotechnology, macrophages can be developed as prominent NP drug carriers. However, there are still many limitations and challenges. First, the sources of autologous macrophages are limited. It is currently impossible to obtain a large number of macrophages through *in vitro* expansion of autologous monocytes derived from patients, while the use of allogeneic macrophages carries a risk of rejection or graft-versus-host reaction. Second, loading NPs into macrophages or anchoring NPs on the surface of macrophages has complex effects on cell function, which remain not fully understood. Third, the local immunosuppressive microenvironment of tumors is closely related to tumor progression; however, there is currently much that is unknown regarding how NPL-Ms regulate the tumor immune microenvironment as well as T cell immune responses. Finally, although the pathways of NP internalization by tumor cells has been extensively studied, our knowledge about the cellular uptake of NPs with various properties by macrophages remains very limited (30, 153). How NP loading affects the function of macrophages in terms of phagocytosis, migration, and immune stimulation must be comprehensively evaluated in future studies. Moreover, although previous studies have shed some light on the possible pathways governing the intracellular trafficking of NPs in macrophages and their release at tumor sites (153, 154), which is depicted in **Figure 2**, precise mechanisms remain largely elusive and await more detailed investigations.

**Table 2:** Macrophage-mediated NP drug delivery in some cancer studies.

NPs	Agents	Macrophage Information	NP Modification	Mechanisms and Features	Cancer Models	REFs
zSOC NPs; NLCs	PTX; DOX	• Raw 264.7 cells	–	• Targeted NP drug delivery	Breast cancer, SUB	(39)
rGO NPs	DOX	• Raw 264.7 cells	PEG-BPEI (PB) coating	• Enhanced NP loading by PB • NIR-triggered DOX release • Combined PTT and CT effects	Prostate cancer, SUB	(102)
NGs; PPy NPs	DOX	• Raw 264.7 cells	Hyaluronic acid (HA) coating	• Enhanced NP loading by HA • NIR-triggered DOX release • Combined PTT and CT effects	Breast cancer, SUB	(119)
AuNSs	–	• Raw 264.7 cells	Surface anionic charging	• Enhanced NP loading • PA imaging and PPT effects	Breast cancer, SUB	(40)
SNPs	DOX	• Raw 264.7 cells • M1 polarization upon NP loading	–	• Effective NP uptake, tumor site homing, and slow drug release • Drug release in exosomes	Glioblastoma, SUB	(151)
LNPs	Sorafenib	• Raw 264.7 cells • M1 polarization by LPS treatment	–	• Enhanced NP tumor site homing • Enhanced targeted drug therapy • Enhanced immune responses	liver cancer, SUB	(24)
AuNSs	–	• Raw 264.7 cells • LPS-treated or -untreated(M1 or M0 type macrophages)	–	• Enhanced NP loading, tumor site homing, and PTT effect by M1 macrophage polarization	Head and neck cancer, SUB, Xenograft	(117)
PLGA NPs	DOX	• Bone marrow-derived macrophage • M1 polarization by LPS and IFN-γ treatment	–	• Effective NP uptake, tumor site homing, and slow drug release • Crossing the BBB to brain tumors	Glioblastoma, orthotopic	(118)
ZnPc NPs	Oxaliplatin prodrug	• Bone marrow-derived macrophages • M1 polarization upon NP loading	–	• Drug release in low-pH sites • Combined PDT and CT effects • Enhanced immune responses	Breast cancer, SUB; Lung metastasis	(41)
Liposomes	DOX	• Primary peritoneal macrophages	–	• Targeted NP drug delivery	Lung cancer, SUB, Xenograft	(93)
PSMA NPs	Mertansine	• Bone marrow-derived Ly6c^high^ inflammatory monocytes	Legumain-sensitive peptide coating	• On-demand drug release by macrophages at l ung metastasis	Lung metastasis of breast cancer	(88)
CPNs	–	• Bone marrow-derived monocytes • Human monocytes THP-1 cells	–	• Crossing the BBB to brain tumors • PDT effects	Glioblastoma, orthotopic	(96)
Liposomes	–	• Human peripheral blood monocytes • Human peritoneal macrophages	Oligomannose coating	• Effective NP loading • Accumulation of the NPL-Ms in peritoneal micrometastatic sites	Gastric cancer metastatic model	(152)
SWNTs	–	• Circulating Ly-6C^high^ monocytes • Cell encapsulation *in vivo*	RGD peptide coating	• NP ligand functionalization • NPL-Ms generation *in vivo* in a selective macrophage subtype	Glioblastoma, SUB	(120)
PLGA NPs	Vincristine	• Circulating monocytes • Cell encapsulation *in vivo*	Binding on damaged RBC membranes	• Enhanced NP drug delivery by a cell relay strategy	Breast cancer, SUB in Rat	(109)
AuNRs	–	• Raw 264.7 cells (*in vitro* encapsulation) • Circulating Ly-6C^high^ monocytes (*in vivo* encapsulation)	CpG coating; Cloaking in apoptotic bodies	• Immune stimulation by CpG • PTT effects	Breast cancer, SUB	(108)

AuNRs, gold nanorods; AuNS, gold nanoshells; BBB, blood–brain barrier; CPNs, conjugated polymer nanoparticles; CT, chemotherapy effects; DOX, doxorubicin; LNPs, lipid nanoparticles; NGs, nanogels; NLCs, nanostructured lipid carriers; OMLs, oligomannose-coated liposomes; PA, photoacoustic; PDT, photodynamic therapy; PLGA, polylactic-co-glycolic acid; PSMA, poly (styrene-co-maleic anhydride); PTT, photothermal therapy; PTX, paclitaxel; rGO, reduced graphene oxide; SNPs, silica-based nanoparticles; SOC, N-Succinyl-N’-octyl chitosan; SUB, subcutaneous tumor model; SWNTs, single-walled carbon nanotubes; ZnPc, photosensitizer zinc phthalocyanine.

## Emerging Concepts and Novel Strategies in Macrophage Engineering

**Figure 3:**
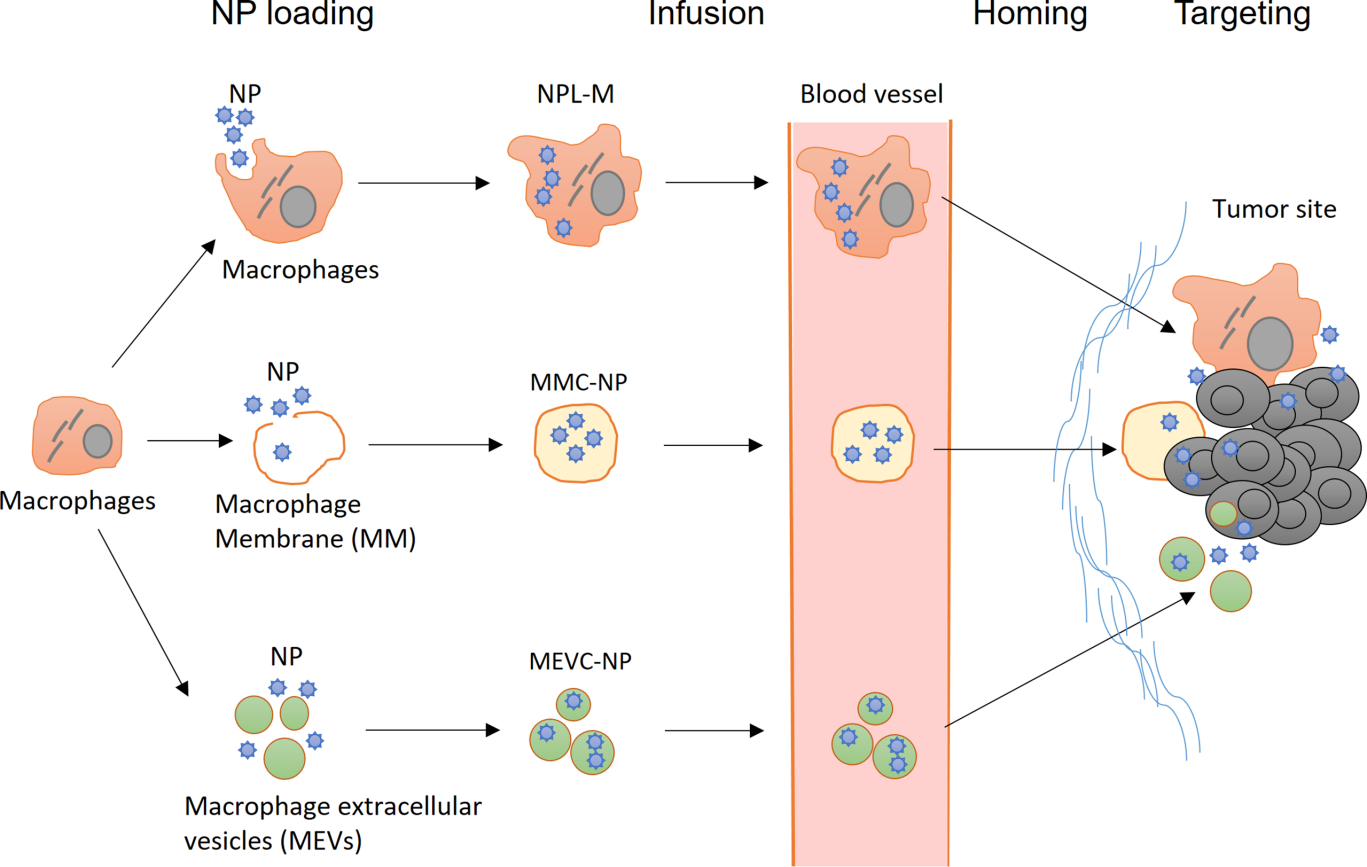
Application of nanotechnology in the engineering of macrophages. (Top) After infusion, NPL-Ms actively migrate to tumor tissue and release NPs locally, resulting in enhanced antitumor effects. (Middle) Macrophage membrane-coated NPs (MMC-NPs) have a prolonged half-life in circulation and a strong affinity at the tumor site for vascular endothelial cells that facilitate their tumor site homing and accumulation. (Bottom) Macrophage-derived extracellular vesicle-coated NPs (MEVC-NPs) can infiltrate tumor sites, where they are taken up by tumor cells, inducing significant cell death.

In recent years, new strategies have emerged in the field of macrophage engineering. For example, macrophage membranes and macrophage extracellular vesicles (MEVs) have been successfully utilized for NP loading; these approaches not only retain some characteristics of macrophages but also greatly expand the compatibility and loading capacity of NPs (**Figure 3**). Another research hotspot involves targeting macrophages with NPs, thereby enhancing the phagocytic function of macrophages and promoting the differentiation of macrophages toward the M1 type. In addition, the success of CAR-T technology has inspired studies of macrophage engineering with CARs for cancer immunotherapy.

### Macrophage Membrane-Coated NPs (MMC-NPs) and Macrophage Extracellular Vesicle-Coated NPs (MEVC-NPs)

In preparing MMC-NPs, the structure of the macrophage cell membrane is disrupted by physical or ultrasonic methods, and then, the cellular contents are removed. After coincubation with NPs, the cell membrane spontaneously closes to form MMC-NPs (155). MMC-NPs have several important advantages. First, the use of the cell membrane eliminates the potential adverse effects of NP loading on the function of macrophages. In addition, it does not cause immune rejection if the autologous cell membrane is used and thus significantly prolongs the half-life of NPs in circulation. Moreover, many macrophage membrane proteins are retained on the surface of MMC-NPs, which may facilitate tumor homing (156). Xuan et al. prepared a macrophage membrane-coated gold nanoshell (AuNS). These MMC-NPs accumulated in tumor sites through the interaction between macrophage membrane molecules and adhesion molecules on the vascular endothelial cells of tumor tissue, leading to significant antitumor effects in a mouse breast cancer model. Compared with NPs coated with erythrocyte membranes, MMC-NPs were more effectively enriched in tumor tissues. In addition, due to the membrane fusion effects, the uptake of MMC-NPs by tumor cells was significantly improved compared to that of free NPs (127). Zhang et al. prepared MMC-NPs loaded with pH-sensitive PTX-NPs. Upon reaching the tumor tissue, these MMC-NPs released PTX-NPs in response to the weakly acidic environment in the tumor stroma; after internalization by the tumor cells, the PTX was quickly dissociated from the PTX-NPs in the highly acidic environment of lysosomes inside the tumor cells and exerted significant anticancer effects in a mouse breast cancer model (125).

Extracellular vesicles (EVs) are cell-derived and membrane-coating particles carrying cell-specific DNA, RNA, and proteins. They are usually divided into three categories based on their size and origin: exosomes (30-150 nm), microvesicles (MVs, 50 nm-1 µm), and apoptotic bodies (50 nm-5 µm) (157). EVs can be efficiently internalized by other cells, mediating the exchange of biological substances between cells and playing important roles in tumor progression (158–160). The potential application of macrophage-derived exosomes and MVs in cancer therapy has attracted great attention recently because of their excellent biocompatibility and high NP-loading capacities (161, 162). Kim et al. found that free PTX coated with M1 macrophage-derived exosomes (PTX-M1-exos) had strong anticancer effects in a mouse model of pulmonary tumor metastases (133). They demonstrated that PTX-M1-exos were more effectively internalized by tumor cells than NPs-PTX, as indicated by the nearly complete colocalization of PTX-M1-exos with cancer cells 4 h after intranasal administration (133).

The communication between tumor cells and macrophages *via* exosomes is believed to play an important role in tumor development (163, 164). Interestingly, tumor cells efficiently take up EVs derived from macrophages (129, 131, 133, 165), although the underlying mechanism is not very clear. It was reported that the acidic tumor microenvironment may promote membrane fusion between exosomes and tumor cells (166). In addition, macrophage-derived exosomes may carry certain cell membrane proteins capable of specifically binding to tumor cells, thus promoting membrane fusion and exosome internalization (167, 168). Moreover, after entering tumor cells, exosomes may alter intracellular transport pathways to prevent their rapid release from tumor cells (169), thus allowing more drugs to enter the cytoplasm and nucleus and exert a more significant therapeutic effect (132).

In addition to improving drug delivery, macrophage-derived EVs also regulate antitumor immune responses. For example, Choo et al. found that exosome-mimetic nanovesicles (M1NVs) derived from M1 macrophages were enriched in tumor tissue after intravenous infusion, which induced the differentiation of TAMs from M2 to M1 macrophages and thus enhanced the effect of anti-PD-1 immunotherapy in tumor-bearing mice (170). Wei et al. found that macrophage-derived microparticles could be preferentially taken up by TAMs in tumor tissues, thereby exerting immunomodulatory effects in tumor-bearing mice (171). Cheng et al. reported that after subcutaneous injection, M1 macrophage-derived exosomes could be taken up by both macrophages and dendritic cells in lymph nodes, where they secreted large amounts of Th1-type cytokines and enhanced antitumor immune responses in a melanoma mouse model (172). In summary, using macrophage membranes or macrophage-derived EVs as carriers can improve drug loading and partially solve the shortage of cell sources. These novel drug carriers can not only target tumor sites but also activate antitumor immune responses and therefore hold great promise in cancer therapy (173–175).

### Targeting TAMs *via* Nanotechnology for Improved Anticancer Activity

As described earlier, reprogramming TAMs from the M2 to M1 differentiation status may be an effective cancer treatment strategy (176, 177). To this end, nanotechnology is very useful. A variety of NP designs were reported to be capable of targeting TAMs specifically and inducing M1 differentiation, leading to potent anticancer activities in preclinical models. For example, given that mannose specifically binds to the CD206 receptor on the surface of M2 macrophages, Zhao et al. prepared mannose-encapsulated NPs containing polyinosinic-polycytidylic acid (poly IC) that are capable of inducing M1 differentiation. NPs are preferentially taken up by M2 macrophages and induce M1 polarization, thereby leading to pronounced antitumor effects (178). Qiang et al. prepared M2-targeting NPs (M2NPs) by coating the NPs with an M2 macrophage-binding peptide and loaded them with small interfering RNA (siRNA) targeting colony-stimulating factor-1 receptor (CSF-1R), which plays a critical role in M2 differentiation. M2NPs effectively targeted M2-type TAMs and induced M1 differentiation, thereby inhibiting the growth of tumors in tumor-bearing mice (179).

In addition, multifunctional NPs can be generated for better treatment outcomes. Zhang et al. constructed NPs containing mesoporous Prussian blue (MPB) with a surface modified by low-molecular-weight hyaluronic acid. After tail vein injection, the NPs selectively accumulated in M2 TAMs in tumors, leading to reprogramming from M2 to M1 macrophages. In addition, the NPs generated oxygen through the catalytic decomposition of endogenous hydrogen peroxide (H_2_O_2_) and thus corrected hypoxia in the tumor microenvironment, acting as *in situ* O2 generators (180). Han et al. loaded NPs with CpG oligodeoxynucleotides (CpG-ODN), baicalin, which has immunomodulatory functions, and the human melanoma antigen Hgp100_25–33_. The NPs were further coated with an RBC membrane carrying galactose that facilitated the targeted delivery of the NPs to TAM by binding galactose-type lectin (Mgl) on the TAM cell surface (181). The results demonstrated that these multifunctional NPs promoted M1 differentiation and enhanced the antigen-specific immune response, thereby exerting a significant antitumor effect in melanoma tumor-bearing mice (181).

CD47 on the tumor cell surface binds to SIRPα on the surface of macrophages, which activates the Src homology region 2 (SH2) domain phosphatases SHP1 and SHP2 and thereby transmits a “don’t eat me” signal to macrophages. Ramesh et al. prepared NPs containing two types of inhibitors: a CSF1-R inhibitor capable of promoting M1 reprogramming and an SHP2 inhibitor that blocks CD47-SIRPα signal transduction and thus enhances phagocytosis. In addition, they coated NPs with anti-CD206 to improve the efficacy of M2-type TAM targeting. The results demonstrated that these multifunctional NPs exerted a significant antitumor effect, mainly through modifying TAMs in breast cancer and melanoma mouse models (182). In addition, the CRISPR/Cas9 gene editing system can also be delivered to macrophages using NPs. Lee et al. used gold NPs to carry the Cas9 protein and sgRNAs targeting the *PTEN* gene. These NPs were mainly phagocytosed by macrophages residing in the liver and spleen after tail vein injection, leading to a gene-editing efficiency of greater than 8% in macrophages (183). Nanotechnology can also be used to transport mRNA or siRNA to a specific cell population in a targeted manner (184, 185). For example, NPs carrying *PTEN* mRNA were effectively delivered to PTEN^null^ cancer cells, and restoration of PTEN expression induced immunogenic death of cancer cells and thus induced potent antitumor immune responses in melanoma tumor-bearing mice (186). In summary, by combining nanotechnology and a variety of approaches, TAMs can be modified in a targeted manner, and their anticancer activities can be promoted.

### Equipping Macrophages With CARs *via* Genetic Manipulation

**Figure 4:**
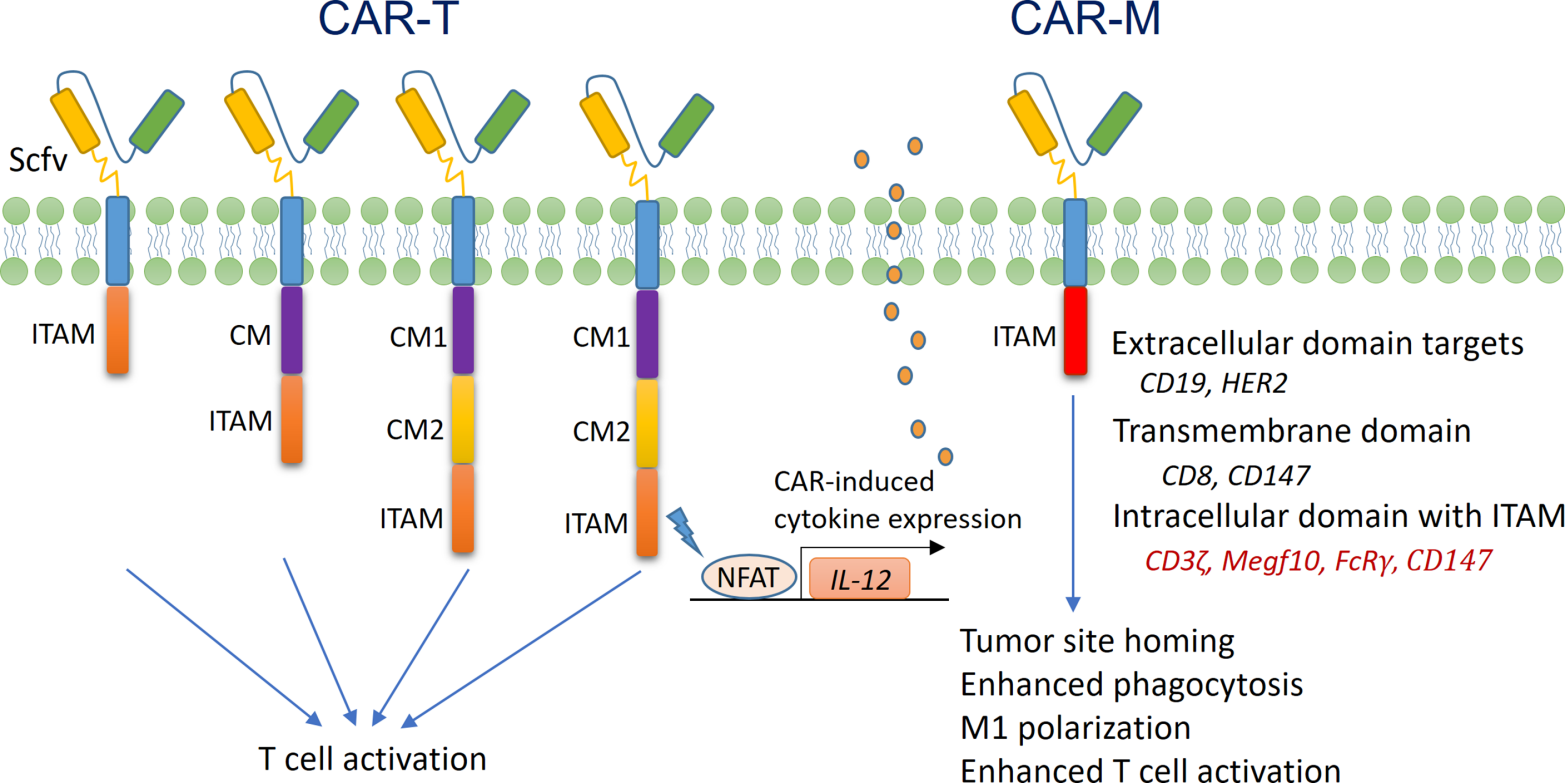
Structure and function of CAR-T cells and CAR-Ms. (Left) The structure of first-generation T cell CARs mainly includes an ScFV extracellular domain that recognizes tumor antigens, a TM domain, and an intracellular domain that contains ITAM and is responsible for signal transduction (usually derived from the intracellular domain of CD3ζ). The structure of second-generation T cell CARs includes an additional intracellular signal transduction domain from costimulatory molecules (CMs), such as CD28 and 4-1BB. The structure of third-generation T cell CARs includes two or more CM domains, which further enhance T cell activation. The structure of fourth-generation CARs includes a nuclear factor of activated T cells (NFAT)-responsive gene expression cassette, which drives the expression of an immunoregulatory gene, such as IL-12. Once CAR-T cells are activated, NFAT translocates to the nucleus and activates the expression of IL-12, thereby promoting anticancer activity. (Right) Currently, the structure of macrophage CARs is based on that of first-generation T cell CARs. The intracellular domain of CD3ζ, FcRγ or Megf10 is used for signal transduction. In addition, CAR-Ms are preferentially fixed at the M1 differentiation status, with enhanced phagocytic and antigen presenting activities.

The concept of CARs was first tested in T cells, and the application of CAR-T cells in the treatment of blood cancers was successful (187, 188). As shown in **Figure 4**, T cell CARs are mainly composed of an extracellular domain of a single-chain variable fragment (Scfv) that specifically recognizes target molecules, a transmembrane (TM) domain, and an intracellular domain responsible for signal transduction. This design confers T cell tumor cell-specific cytotoxicity in an MHC-independent manner. However, to date, CAR-T therapy has have a limited effect in solid tumors (187, 189), and researchers have begun to ask whether CAR-modified macrophages (CAR-Ms) could be useful in cancer therapy. It is known that the “eat me” signal molecules on tumor cells, such as lipid phosphatidylserine (PS), are recognized by corresponding scavenger receptors on macrophages, resulting in the activation of phagocytosis (190, 191). In addition, Fcγ receptors (FcγRs) on macrophages mediate antibody-dependent cellular phagocytosis (ADCP) by binding to the Fc segment of the IgG antibody (190, 191). The basic structures of these abovementioned phagocytic receptors all include an extracellular domain, a TM domain, and an intracellular domain, similar to those of CAR molecules. Ligation of the extracellular domains of these receptors induces phosphorylation of tyrosine in the immunoreceptor tyrosine-based activation motif (ITAM) of the intracellular domain of these receptors, leading to cytoskeletal and membrane remodeling events that promote the ingestion of tumor cells by macrophages (192).

A series of recent studies have demonstrated that the antitumor activity of macrophages can be enhanced by modifying phagocytic receptors with CAR technology (193–196). Morrissey et al. prepared mouse CAR-Ms by lentiviral transduction. The extracellular domain of the CAR recognized CD19, and the TM domain was derived from CD8 (194). They found that the intracellular domains from either Megf10 or FcRγ molecules were able to mediate the specific phagocytosis of CD19-expressing Raji B cells by the CAR-Ms. Interestingly, replacement of the intracellular domain with that of CD3ζ (which contained three ITAMs and had high homology with FcRγ) achieved a similar effect (194). Klichinsky et al. prepared CAR-Ms with human peripheral blood monocytes. The CAR molecules had an extracellular domain that recognized human epidermal growth factor receptor 2 (HER2) and an intracellular signal domain from CD3ζ (193). The CAR-Ms were able to specifically recognize and phagocytose HER2^+^ tumor cells, and a single-dose infusion of the CAR-Ms significantly inhibited the growth of HER2^+^ xenograft tumors. Importantly, after infusion, the CAR-Ms accumulated in liver and tumor tissues and survived *in vivo* for at least 2 months (193). In the preparation of CAR-Ms, delivering CAR genes into macrophages is technically challenging. The authors demonstrated that a replication-incompetent chimeric adenoviral vector (Ad5f435) not only efficiently transferred the CAR genes into macrophages but also induced M1 differentiation. Such CAR-Ms activated CD4+ Th1 cells and, more importantly, CD8+ cytotoxic T cells through cross-presentation, thereby promoting a strong antitumor effect (193). Zhang et al. prepared CAR-Ms to target the extracellular matrix rather than tumor cells, with the aim of enhancing immune infiltration into solid tumors (195). The TM and intracellular domains of the CAR molecules were all derived from CD147, which drives the expression of matrix metalloproteinases (MMPs) in macrophages. The CAR-Ms were detected in tumor tissues 24 h after tail vein injection, and their numbers peaked at 3 d, during which time the collagen content in the tumor stroma was significantly decreased due to the increased activity of MMPs. Further analysis revealed that the anticancer effect of the CAR-Ms in tumor-bearing mice was associated with increased CD3^+^ T cell infiltration (195).

CAR-M technology holds great potential for the treatment of solid tumors. However, at present, this field is still in its infancy, and there are many challenges. For example, most solid tumors lack suitable tumor-specific antigens for CAR design. In addition, the impact of different TM domains and intracellular domains on the function of CAR-Ms remains unclear. In the clinical application of CAR-T cells, cytokine release syndrome (CRS) and immune effector cell-associated neurotoxicity syndrome (ICANS) are the two most serious side effects, both of which may be related to excessive inflammatory cytokines derived from CAR-T cells (197). A recent study utilized the intracellular domain of the MERTK kinase to develop CAR-Ms. These CAR-Ms effectively eliminated SARS-CoV-2 virus *in vitro* by enhanced phagocytosis without upregulation of proinflammatory cytokine expression (198). Such results indicate that it is possible to optimize the design of CAR-Ms to reduce their potential side effects. In the context of cancer therapy, inducing M1 differentiation may be preferred, as it can improve the phagocytic activity of CAR-Ms; however, such manipulation may have unpredictable side effects and needs to be carefully evaluated using preclinical models.

## Conclusion and Perspective

Macrophages are extremely versatile and possess a variety of antitumor properties. They can kill tumor cells directly by phagocytosis or indirectly by activating other immune cells. However, in the tumor microenvironment, their antitumor activities are often inhibited (192). With the rapid development of nanotechnology and transgenic technology, engineering macrophages has become an important research direction in cancer therapy (199). Numerous studies have demonstrated that engineered macrophages can actively migrate to tumor tissues and kill tumor cells effectively. However, they can also migrate to normal tissues and organs after infusion. Considering the relatively long lifespan of these cells, their migration, distribution, and potential toxicity to normal tissues needs to be closely monitored *in vivo*, and novel techniques such as macrophage imaging might be useful in this regard (193, 200). It is of great significance to investigate how to better control the migration of engineered macrophages to reduce their accumulation in normal tissues. Studies have shown that chemotherapy, radiotherapy, and immunotherapy (such as STING agonist treatment) can all stimulate inflammation to a certain extent, thereby transforming cold tumors into hot tumors (201–203). Such transformations could improve the directional migration of engineered macrophages to tumor sites, thus enhancing their therapeutic effects while reducing potential off-target or on-target toxicities.

Notably, when NPs or macrophage membrane-coated NPs are used to deliver genetic materials into macrophages, including DNA, mRNA, noncoding RNA, and the CRISPR system, the efficacy of genetic modification seems to be greatly improved (25, 204–206). However, at present, our understanding of the interactions between these gene carriers and macrophages, in terms of phagocytosis, transport, and release, is very limited, and further investigation is needed. In addition, after engineered macrophages enter tumors, their activities may be antagonized by local TAMs that are usually immunosuppressive; therefore, conducting in-depth studies is important to determine whether the pre-existing TAMs will significantly impact the function of engineered macrophages, or vice versa. In this regard, methods for local TAM depletion can be used in sequential combination with engineered macrophages (207, 208), i.e., disruption of the immunosuppressive microenvironment dominated by depleting TAMs followed by activation of antitumor immune responses by supplying engineered macrophages.

Reprogramming macrophages from M2 to M1 polarization can be achieved through various means, such as by using IL-12, CD40 agonists, or CSF-1R inhibitors (209–211). In addition, “don’t eat me” molecules, such as CD47 and MHC-I, on tumor cells inhibit the phagocytic function of macrophages by binding SIRPα or LILRB1, respectively, on macrophages (64, 212). Therefore, interference with these “don’t eat me” molecules may further enhance phagocytosis by engineered macrophages. These methods could further promote the anticancer activities of engineered macrophages. Finally, if needed, methods of TAM depletion *in vivo* can serve as a safeguard to remove engineered macrophages that have serious side effects.

## Author Contributions

Initial manuscript writing, XD, LW, YL, GY, and XZ. Revision and editing, XS, HC, YZ, DZ, GY, and XZ. Figure drawing, YZ and XZ. Funding acquisition, XD, GY, and XZ.

## Funding

This work is supported by the National Natural Science Foundation of China (81771681, 82172931, and 32170915), Jiangsu Major Disease Biological Resource Foundation (SBK202004006), Jiangsu Health Committee Research Project (M2020076), Wu Jieping Medical Foundation (320.6750.19088-89), and the Nantong Science and Technology Project (JCZ19102).

## Conflict of Interest

The authors declare that the research was conducted in the absence of any commercial or financial relationships that could be construed as a potential conflict of interest.

## Publisher’s Note

All claims expressed in this article are solely those of the authors and do not necessarily represent those of their affiliated organizations, or those of the publisher, the editors and the reviewers. Any product that may be evaluated in this article, or claim that may be made by its manufacturer, is not guaranteed or endorsed by the publisher.
